# Improving the knowledge of high school students about zoonotic diseases from pets in Medellín-Colombia

**DOI:** 10.14202/vetworld.2021.3091-3098

**Published:** 2021-12-09

**Authors:** Natalia Uribe Corrales, Karen Velásquez Giraldo, Catalina María Saldarriaga Garcés, Angie Lorena Navarro Giraldo

**Affiliations:** Department of Agricultural Sciences, Faculty of Veterinary Medicine, Lasallian University Corporation (Unilasallista), Caldas, Antioquia, Colombia

**Keywords:** cats, diseases, dogs, education, zoonoses

## Abstract

**Background and Aim:**

The “One Health” concept is a global strategy that recognizes that public health is related to animal health and the environment; however, the role of domestic animals and their involvement in the transmission of zoonoses is often underestimated. The aim of the study was to evaluate and improve the knowledge about zoonotic diseases of domestic animals in high school students from Medellín, Colombia.

**Materials and Methods:**

A quasi-experimental intra-subject study was carried out. This study was conducted with 11^th^-grade students from four schools in Medellín, Colombia. A structured multiple-choice questionnaire was used from March 2021 to May 2021. The research had two phases, first, “naive” knowledge and learning. Then, descriptive, association, and comparative analysis were carried out using absolute and relative frequencies, Pearson’s Chi-square test, and MacNemar’s test with a value of p<0.05 considered statistically significant.

**Results:**

A research poll from 315 students of four private schools found that feeding their pets with raw food and leftovers cooked for human consumption were common practices; the results also show a lack of knowledge of their pets’ immunization deworming status. It was understood that when the students were able to identify at least two symptoms of zoonoses, one route of its’ transmission and two preventive measures, we found that only 12.49% of the polled students had proper knowledge of the disease in domestic animals. After conducting an educational strategy, the findings show a general increase in knowledge, leading us to accept that the academic approach was adequate to statistically increase the recognition of symptoms, routes of transmission and preventive measures (p=0.00).

**Conclusion:**

The use of the theoretical lecture is effective to improve the understanding of the concept of transmission of diseases from animals to humans; in addition, the results show an increase of knowledge in high school students of the related symptoms, transmission routes, and preventive measures of zoonoses diseases in the region.

## Introduction

The “One Health” concept is a theoretical lecture strategy that recognizes that public health in humans is related to animal health and the environment. It requires the collaboration of physicians, veterinarians, environmental scientists, public health professionals, and wildlife experts; however, even knowing domestic animals can increase the presence of some diseases in humans, many times, the interaction of domestic animals is often underestimated [1,2]. During past decades, dogs and cats often spent a good part of their lives indoors in close contact with their owners. As a result, several zoonotic infectious diseases may be transmitted directly or indirectly from these species; thus, the World Small Animal Veterinary Association considered that there are three critical areas of “One Health” regarding domestic animals: The human-domestic animal bond, comparative medicine, and zoonotic infectious disease [2-4], therefore, the lack of awareness about zoonotic diseases and the role that pets can play with their interaction with humans, are some of the most important reasons for the outbreak of zoonotic diseases in people, improving awareness among the community should also improve the prevention and control of those diseases [5].

Regarding some interaction practices, some authors have shown that 50% of owners allow pets to lick their faces; 60% of the pets visit the bedroom; 45-60% (dogs-cats) are allowed on the bed; 18-30% (dogs-cats) sleep with their owner in bed and, among the cats, and 45% are allowed to jump onto the kitchen sink [6]. Those practices could be potential exposure to pathogens, reminding that animals can transmit microorganisms through direct or indirect contact through biting, licking, scratching, sneezing, coughing, handling body fluids, secretions, or contaminated bedding [7].

Other risk factors for infection include lack of regular and efficient deworming, absence of routine vaccination programs, poor hygiene practices, low socio-economic and educative factors, failure to regularly pick up and dispose of feces, lack of pets’ population control measures, and consequent high numbers of free-ranging dog and cat populations [8]. Many of those factors can be mitigated by simple measures, such as hand hygiene and modification of animal contact behaviors. Therefore, being aware of the risks of getting a zoonotic disease and the measures that can reduce the risk are requirements that must be met [8,9].

At present, many studies have evaluated the knowledge, attitude, and practices of the public toward pet ownership and associated zoonoses [10-14]; nonetheless, few studies show what the knowledge that students have related to zoonoses is. According to some authors, the student population is vulnerable to these diseases due to ignorance and confusion about what these diseases are (transmission mechanisms, effects on the human being’s health, and the preventive measures), which increases the odds of getting sick [15].

Therefore, this study aimed to evaluate and improve the knowledge about zoonotic diseases of domestic animals in high school students from Medellín, Colombia.

## Materials and Methods

### Ethical approval

The Ethics Committee for Experimentation with Animals of the Lasallian University Corporation has approved this research.

### Study period and area

**Figure-1:**
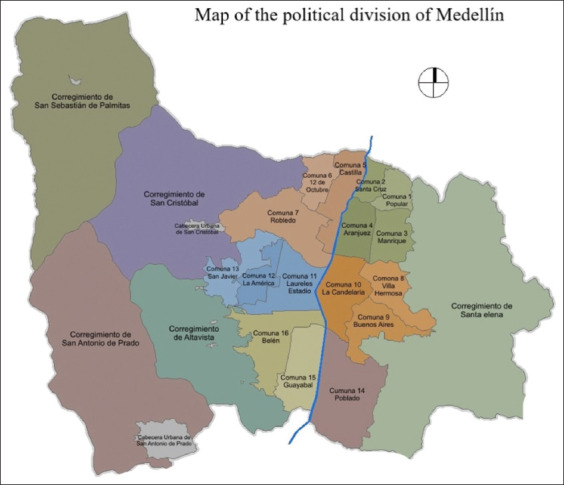
Map of Medellín [Source; https://commons.wikimedia.org/wiki/File: Corregimientos_de_Medellin.svg].

The study was conducted from March 2021 to May 2021. Medellín is a Colombian municipality, capital of the region of Antioquia. It is the second most populated city in the country after Bogotá. In the widest part of the natural region known as Aburrá Valley, it is in the central Andes Mountain range. It extends on both banks of the Medellín River, which crosses it from south to north, and is the main nucleus of the metropolitan area of the Aburrá Valley [16]. The city had a population of 2,533,424 inhabitants during the year 2020. The latitude and altitude of the city result in a subtropical monsoon climate. The climate is temperate and humid, with an average temperature of 21.6°C [16,18] (Figure-1).

#### Study design

A quasi-experimental intra-subject study was conducted using the One Health approach. This study was carried out with 11^th^-grade students from four schools located in Medellín, Colombia.

#### Sampling

No sampling was carried out but instead worked with all the students who wanted to participate, who did not wish to participate, in the same way, we provided the educational component.

#### Questionnaire

A structured multiple-choice questionnaire was used for this study; it was written in Spanish. Thirty questionnaires had been pre-tested to assess the suitability of questions (written and multiple-choice answers). The final format took approximately 15-20 min to complete. The questionnaire consisted of 42 questions, divided into three sections: (i) Family structure (ii) animal species primary care, (iii) Zoonoses (symptoms, transmission mechanism, and preventive measures).

#### Research process

The research had two phases.

Phase 1 “Naive” knowledge the teachers at the schools provided the questionnaire link to the students without giving them an introductory lecture on the questionnaire’s topics to assess the “prior knowledge” of the adolescents.

Phase 2 learning: Students attended a theoretical lecture session. The session consisted of 120 min, by a virtual approach. We provided educational posters, slides, comics, and video clips with relevant content and messages [19]. Two weeks after the lecture, the same questionnaire of Phase 1 was again administered to assess the efficacy of the educational strategy and the students’ learning rate. Cronbach’s alpha for the 42-item knowledge scale before and after the intervention was 0.73 and 0.75, respectively.

For knowledge assessment of the students, we asked about some endemic or historically critical zoonotic diseases in the country, such as rabies, brucellosis, leptospirosis, bartonellosis, leishmaniasis, giardiasis, toxoplasmosis, toxocariasis, ancylostomiasis, scabies, and dermatophytosis; the proper knowledge was only considered “correct” if the students were able to correctly identify at least two signs for each disease, one transmission mechanism, and at least two preventive measures.

### Statistical analysis

Relative frequencies summarized demographic, basal knowledge, attitudes, and practices data. We use Pearson’s Chi-square test to detect an association between demographic, attitudes, and practices variables and zoonoses knowledge; and MacNemar test to compare the baseline to the post-education intervention line, with a p<0.05 regarded as statistically significant. Analyses were performed with Stata® software version 15 (StataCorp LLC, TX, USA).

## Results

We researched four private schools with a total of 315 students. Overall, the participants were mainly men; 82.22% were primarily from the poor or middle class, 78.41% had a pet, and the most common was a dog 50.79%. For both (dogs and cats), males were primarily entire 50.53% and 61.90%, although females were predominantly spayed 56.71% and 77.27%, respectively. In addition, 95.23% of the respondents said they had heard the term zoonoses. The detailed demographic profile of the participants is presented in Table-1.

**Table 1:** Frequency table for demographic and pets’ variables.

Variable	Category	Frequency	Percentage
Student Gender	Male	202	64.13
	Female	113	35.87
Home location	Rural	124	39.37
	Urban	191	60.63
Economic status	Poor	137	43.49
	Middle	122	38.73
	High	56	17.78
Do you have a pet?	Yes	247	78.41
	No	68	21.59
What kind of pet do you have?	Canine	160	50.79
	Feline	87	27.62
	Not have a pet	68	21.59
Where did you get your pet?	Refugees	110	44.53
	Animal Store	95	38.46
	It was a gift	42	17.00
What is your canine’s gender?	Male	93	58.12
	Female	67	41.87
What is your feline’s gender?	Male	21	24.13
	Female	66	75.86
What is your canine’s reproductive status?	Entire male	47	50.53
	Neutered male	46	49.46
	Entire female	29	43.28
	Spayed female	38	56.71
What is your feline’s reproductive status?	Entire male	13	61.90
	Neutered male	8	38.09
	Entire female	15	22.72
	Spayed female	51	77.27
Have you ever heard the term zoonoses?	Yes	300	95.23
	No	15	4.76

Relating to practices, 35% of the dogs are fed with raw or table scrap. An average of 39.49% of the students did not know how often their pets had been vaccinated or dewormed. Regarding fleas or ticks, 81.25% of the students reported seeing them on their dogs and 67.82% on their cats. The detailed knowledge and practices of the participants are presented in Table-2.

**Table 2:** Knowledge and practices of the students on pet farming.

Variable	Specie	Category	Frequency	Percentage
What do you feed your pet?	Canine	Raw	16	10.00
		Table scrap/human food	40	25.00
		Commercial food	104	65.00
	Feline	Commercial food	56	64.37
		Table scrap/human food	31	35.63
See a veterinarian regularly (at least once a year)?	Canine	Yes	98	61.25
		No	62	38.75
	Feline	Yes	52	59.77
		No	35	40.23
How often is your pet vaccinated?	Canine	Annually	98	61.25
		Do not Know	62	38.75
	Feline	Annually	52	59.77
		Do not Know	35	40.23
How often does your pet deworm?	Canine	Annually	69	43.13
		Every 3 or 4 months	29	18.13
		Do not remember	62	38.75
	Feline	Every 3 or 4 months	52	59.77
		Do not know	35	40.23
Have you seen fleas or ticks on your pet?	Canine	Yes	130	81.25
		No	30	18.75
	Feline	Yes	59	67.82
		No	28	32.18

In addition, 72.28% of the students had experienced mouth contact with their pets; 64.36% reported contact with pet feces. Finally, we found that 62.38% washed their hands after having contact with their pets, although just 55.56% of them do that consistently; 37.62% never washed their hands after having contact with their pets (Table-3).

**Table 3:** Sanitation and pet contact-related attitudes of the respondents.

Variable	Category	Frequency	Percentage
Having mouth contact with any part of your pet	Yes	228	72.28
	No	87	27.72
Touching or having any contact with your pet faces	Yes	203	64.36
	No	112	35.64
Do you (any member of the family) wash their hands after touching your pet?	Yes	196	62.38
	No	119	37.62
How often	Always	28	14.29
	Usually	59	30.16
	Sometimes	109	55.56

**Figure-2:**
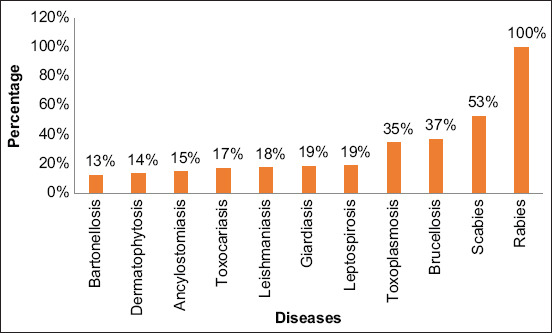
Percentage of students who had heard about different zoonotic diseases in Phase 1.

**Figure-3:**
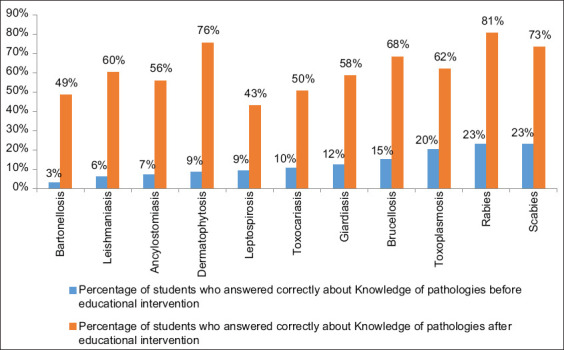
Knowledge before and after educational intervention from 315 students in Medellín.

At Phase 1, 100% of the students had heard about rabies; however, regarding other diseases, a low percentage of the participants had heard about them (Figure-2). Although a low percentage of students had heard about some diseases, the knowledge about symptoms, route of transmission, and preventive measures was lower. Scabies, rabies, and toxoplasmosis were the conditions where they showed more ability 23.01%, 22.70%, and 20.31%, respectively (Figure-3). We did not find any association between demographic, attitudes, and practices variables and zoonoses knowledge.

After the lecture, global knowledge increased to an average of 61.48%, being the highest in rabies (80.63%) and the lowest in leptospirosis (42.86%). Dermatophytosis was the disease that achieved the most significant increase of knowledge, 67.32%, followed by rabies and leishmaniasis, the ability increased by 57.93% and 54.43%, respectively. As shown in Figure-3 and Table-4, the educational strategy effectively improved their knowledge in recognizing symptoms, route of transmission, and preventive measures in all the conditions. Nonetheless, a substantial proportion of the respondents still did not have the proper knowledge.

**Table 4:** Improvement of the knowledge about zoonotic diseases from pet in high school students.

Knowledge of zoonoses after educational intervention	Knowledge of zoonoses before educational intervention			McNemar’s chi^2^	p-value	Odds ratio	95% Confidence interval

	With knowledge	Without knowledge	Total				
Rabies							
With knowledge	35	219	254	204.45	0.00	43.8	15.15-205.44
Without knowledge	5	56	61				
Total	40	275	315				
Brucellosis							
With knowledge	46	169	215	169.00	0.00	38.5	31.39-69.75
Without knowledge	1	99	100				
Total	47	268	315				
Leptospirosis							
With knowledge	25	110	135	70.13	0.00	6.87	3.51-15.14
Without knowledge	16	164	180				
Total	41	274	315				
Bartonellosis							
With knowledge	37	116	153	101.83	0.00	23.20	7.88-109.88
Without knowledge	5	157	162				
Total	42	273	315				
Leishmaniasis							
With knowledge	44	146	190	66.48	0.00	4.05	2.51-6.82
Without knowledge	36	89	125				
Total	80	235	315				
Giardiasis							
With knowledge	88	96	184	84.64	0.00	24.00	7.28-146.02
Without knowledge	4	127	131				
Total	92	223	315				
Toxoplasmosis							
With knowledge	56	139	195	116.74	0.00	17.37	7.20-55.30
Without knowledge	8	112	120				
Total	64	251	315				
Toxocariasis							
With knowledge	24	135	159	110.25	0.00	15.00	6.48-44.18
Without knowledge	9	147	156				
Total	33	282	315				
Ancylostomiasis							
With knowledge	57	119	176	89.72	0.00	10.81	4.97-28.41
Without knowledge	11	128	139				
Total	68	247	315				
Scabies							
With knowledge	86	149	235	102.76	0.00	8.27	4.43-17.21
Without knowledge	18	62	80				
Total	104	211	315				
Dermatophytosis							
With knowledge	80	158	238	127.86	0.00	14.36	6.68-37.44
Without knowledge	11	66	77				
Total	91	224	315				

## Discussion

It is known that pets have a positive effect on human health. However, the human-pet bond faces many challenges that increase the transmission of infection between pets and humans. It is mainly due to the increasing contact between humans and pets and pathogens secreted by animals in the shared environment. Thus, animals can spread more than 6 of every 10 known infectious diseases, and 3 of every 4 new or emerging infectious diseases in people come from animals [20-23].

The Naïve Knowledge of adolescents shows that they have a significant lack of knowledge in understanding zoonoses. This study found that had heard about zoonotic was higher than previous studies (95.23%). In Ethiopia, the information shows that 76.8% of high school students had heard about zoonoses [24]. In Malaysia, the 11% [25], in a previous study in Nariño, Colombia, and Usme, the authors found that 63% and 74.6% of the respondent had heard about the term zoonoses [26,27], in Austria, Germany, Slovenia, Mauritius, and Japan 67.84% of adolescents know the meaning of the term zoonoses [28]. However, in Brazil, only 28.2% had heard the term zoonosis [29].

In addition, an average of knowledge about zoonoses was found (12.49%). The lower cognitive degree in diseases caused by bacteria was related to *Bartonella* (3.33%) and *Leptospira* (9.13%). Parasite diseases such as *Leishmania* (5.89%) and *Ancylostoma* (6.89%) also had lower cognitive degrees. The higher degree of knowledge was (23.01%) for scabies and rabies (22.70%), like other authors in which the main zoonosis recognized by the students was rabies. A high proportion did not identify leishmaniasis, toxoplasmosis as zoonoses [10,13,30]. This situation is bothering and indicates that most of the students have a low or medium knowledge relating to zoonotic diseases [10,12,13,15,19,30,31], being worrying considering that zoonoses are a reality in the population, and lack of basic knowledge about these diseases is due to the scarce delivery of information to communities [20,23,32-34].

After the lecture, the global knowledge increased to an average of 61.48%, being the highest in rabies (80.63%). Some studies show that health education and hygiene instruction are often underestimated, especially with children and adolescents. However, some authors have shown that health education is almost sufficient to avoid zoonoses such as toxocariasis and others [26,27,35-37].

Regarding practices and attitudes performed by the students, this study revealed that 38.75% of students who have a dog do not take their dog to veterinary service regularly, nor the 40.23% of the students who have a cat. This result indicates that most pet owners’ in Medellín give little care for their pet’s health. In addition, some respondents fail to vaccinate and deworm their animals, presumably since they do not have enough money to go to the veterinarian for preventive check or invest in preventative measures.

In addition, 35% of the respondents fed their dogs with raw food or table scrap food. This result is higher than in Canada, which reported that only 28% of dog owners fed raw food like eggs, meat, or animal products [38]. Still, our results are lower than in Ethiopia, in which 92.1% of the dog owners reported this practice [12]. This difference is mainly due to differences in the economic status of pet owners in countries such as Colombia or Ethiopia.

In addition, we found that some practices such as having mouth contact with the animals, touching pet feces, and do not always wash their hands after contact with their pets are similar to results found in Ethiopia [39], where 14.9% of high school students haring the same house with animals; 24.2% feed their pets with raw food, and 16.7% did not vaccinate their pets; and in Italy, Austria, Germany, Slovenia, Mauritius, and Japan almost 24% of the students are unaware that pets must be dewormed [28], those results exposes them to a tangible risk of being infected by zoonotic parasitic diseases such as *Giardia* or *Toxocara*.

Hence, continuous community motivation, better education, and governmental officials’ awareness about zoonoses are crucial to improving general knowledge because pets can transmit different diseases by infected saliva, contaminated urine or feces, or direct contact [40].

Hand hygiene plays an essential role in reducing the risk of zoonotic infections. In this study, the reported hand washing after having direct contact with the pet was moderate 62.38%. In contrast to this finding, most pet owners in developed countries wash their hands less frequently after contacting their pets. For example, in the United States, 45% of the people reported washing their hands after contact with pets [41]; in the Netherlands, said that 50% of dog owners washed their hands after having contact with their dogs, and only 15% of dog owners in Cheshire, England [6,7]. One possible explanation for this difference could be as most pet owners in developed countries give much attention to farming, sanitation, and the health of their pets.

As awareness of the risk of zoonotic diseases is a prerequisite for effective prevention, the public’s limited knowledge of zoonotic illness is a serious concern. The little understanding of zoonotic diseases in the current study is not surprising because, unfortunately, these topics are not part of the curriculums of high schools in Colombia. Therefore, it is the responsibility of veterinarians to educate pet owners about the importance of properly property their pets and implementing recommended hygiene measures (i.e., avoiding raw pet food, mouth contact, and not washing hands). The One Health concept concerning zoonoses and pets is clear about why veterinarians, physicians, and public health authorities need to work together to ensure that all decisions and implemented measures have an impact on the health of humans, animals, and the environment [2,42,43].

## Conclusion

We observed a positive impact mainly on the knowledge of the symptoms, transmission routes, and preventive measures of zoonotic disease. However, despite meeting the proposed objectives, some high school students still do not identify some zoonoses, so it is essential to emphasize zoonoses that the population is exposed to in the curriculum of the high schools. This finding should warn city managers to develop campaigns to educate the general community and reduce zoonoses’ progression.

The usefulness of the theoretical lecture has its effectiveness in understanding the concept of the transmission of diseases that can transmit animals to humans. In this case, only one academic virtual class was enough to increase the knowledge related to symptoms, transmission mechanisms, and preventive measures for zoonotic diseases from pets.

### Limitations of the study

The study was based on four schools that allowed us to share our knowledge with the students. Preferably, it would have been better if the pandemic situation permitted to increase the sample size in other schools and make the lecture and activities face to face to improve the knowledge of the high school students.

## Authors’ Contributions

NUC: Conceptualization, formal analysis, methodology, and drafted and revised the manuscript. KVG, CMSG, and ALNG: Educative intervention. NUC, KVG, CMSG, and ALNG: Investigation and drafted the manuscript. All authors read and approved the final manuscript.
